# Trends in incident hemodialysis access and mortality in China: results from China Renal Data System 2000–2024

**DOI:** 10.1080/0886022X.2026.2747546

**Published:** 2026-10-25

**Authors:** Wenyuan Gan, Fan Zhu, Xingruo Zeng, Wenli Chen, Huihui Mao, Danni Shao, Licong Su, Peiyan Zhan

**Affiliations:** ^a^ Department of Nephrology, The Central Hospital of Wuhan, Tongji Medical College, Huazhong University of Science and Technology, Wuhan, China; ^b^ Key Laboratory for Molecular Diagnosis of Hubei Province, The Central Hospital of Wuhan, Tongji Medical College, Huazhong University of Science and Technology, Wuhan, China; ^c^ Division of Nephrology, Nanfang Hospital, Southern Medical University, National Clinical Research Center for Kidney Disease, State Key Laboratory of Multi-organ Injury Prevention and Treatment, Guangdong Provincial Institute of Nephrology, Guangdong Provincial Key Laboratory of Renal Failure Research, Guangzhou, China; ^d^ Department of Geriatrics, The Central Hospital of Wuhan, Tongji Medical College, Huazhong University of Science and Technology, Wuhan, China

**Keywords:** Incident hemodialysis patients, vascular access, epidemiological survey, mortality

## Abstract

**Backgrounds:**

Large‑scale multicenter data on vascular access among Chinese incident hemodialysis (HD) patients are limited. We explored temporal trends of initial vascular access and its relationship with all‑cause mortality.

**Methods:**

This retrospective cohort study was conducted using the China Renal Data System, enrolling incident HD patients from January 2000 to April 2024. Vascular access trends and distribution at HD initiation were analyzed.

**Results:**

16,717 patients were enrolled (mean age 55.62 ± 10.93 years; 61.54% male; mean follow‑up 64.41 months). At HD initiation, 29.17% of patients used arteriovenous fistula/graft (AVF/AVG), while 70.83% used central venous catheter (CVC), comprising 60.78% with a maturing AVF/AVG and 10.06% with CVC alone. AVF/AVG use increased from 26.24% in 2015 to 35.77% in 2024, while CVC-only use decreased from 16.16% to 8.27%. Mortality rates per 100 person-years were 4.15 (95% confidence interval [CI] 3.93 – 4.38), 7.24 (95% CI 7.00 – 7.49), and 15.08 (95% CI 14.07 – 16.15) in the AVF/AVG, CVC with maturing AVF/AVG, and CVC-only groups, respectively. In the adjusted Cox model, patients using CVC with a maturing AVF/AVG or CVC alone exhibited 36% and 91% higher all-cause mortality risks, respectively, compared with those using AVF/AVG.

**Conclusions:**

Over the 25-year period, initial vascular access among Chinese incident HD patients gradually shifted from CVC to AVF/AVG, reflecting ongoing and considerable improvements in vascular access practice in China. CVC use at HD initiation was associated with higher all-cause mortality compared with AVF/AVG. These findings support early AVF/AVG creation in end-stage kidney disease patients to avoid CVC use at HD initiation.

## Introduction

The number of hemodialysis (HD) patients in China has been increasing year by year, and this upward trend is expected to continue in the years ahead [1]. Vascular access is vital for HD and is widely recognized as patients’ lifeline. Furthermore, the type of vascular access constitutes a critical determinant of patients’ long‑term prognosis [2]. HD catheters, which emerged in the 1980s [3], are associated with significantly higher risks of infections, thrombosis, hospitalization, and mortality, as well as greater healthcare expenditure, compared with permanent vascular accesses such as arteriovenous fistula (AVF) [3–7]. Accordingly, over the past 20 years, permanent vascular access has remained the most preferred and recommended modality, and is widely recognized as the first choice for most prevalent HD patients. In 1997, the AVF prevalence in the United States was merely 24%. Subsequently, AVF use in the US increased from 32% in 2001 to 63% in 2016. In the United Kingdom, central venous catheter (CVC) prevalence decreased from 28% to 16% between 2011 and 2014, while AVF use in Japan increased to 91% in 2013 [8–10]. Although the AVF remains the predominant vascular access type among prevalent HD patients, approximately two-thirds of incident HD patients still initiate HD with a CVC, whereas fewer than one-third initiate HD with an AVF [11–13]. Initiating HD *via* permanent vascular access may improve the prognosis for the majority of incident HD patients [14], yet inconsistent findings still exist in specific subgroups [13,15]. Over the past two decades, the characteristics of HD patients has changed, with a trend toward advanced age and a higher comorbidity burden [16]. In light of these shifts in the clinical profiles of HD patients, the 2019 Kidney Disease Outcomes Quality Initiative (KDOQI) Vascular Access Guidelines [4] advocate a patient‑centered approach to vascular access selection to optimize access patency, minimize complications, and improve clinical outcomes. Furthermore, the guidelines emphasize that current evidence is insufficient to make recommendations regarding the association between initial vascular access types and all‑cause mortality among incident HD patients.

China has a large population of patients with end-stage kidney disease (ESKD). With a vast territory and unbalanced economic development, it differs significantly from developed countries in ethnicity, economic conditions, medical insurance policies, and clinical practice patterns [17]. Unfortunately, large-scale nationwide multicenter studies on vascular access among incident HD patients are still lacking in China, resulting in insufficient understanding of the trends over time, distribution patterns, and prognostic impacts of vascular access, particularly in special patient subgroups. To address this issue, based on data from the China Renal Data System (CRDS) database, we aimed to investigate the temporal trends and distribution patterns of vascular access as well as its association with mortality among incident HD patients in China, provide insights for optimizing clinical management and improving patient prognosis, and facilitate the exchange of real-world data across countries and regions.

## Methods

### Study design and data source

This multicenter retrospective cohort study aimed to investigate the epidemiological characteristics of vascular access and its impact on mortality among incident HD patients in China, using de-identified data collected from the CRDS database between January 2000 and April 2024 (http://www.crds-network.org.cn/#/). The CRDS database is a joint initiative of the National Clinical Research Center for Kidney Disease and the China Center for Disease Control and Prevention, and formed by the regional medical centers across China. All of the laboratories of the participating centers had passed the annual External Quality Assessment by the Chinese National Center for Clinical Laboratories. The CRDS databases includes data of more than 10 million patients from 35 large urban academic centers that cover the major geographic regions across China. The accuracy and completeness of this database have been verified in our previous studies and by other validation activities [18,19].

### Study population and clinical variables

We identified a cohort of patients from the CRDS database who initiated HD between January 2000 and April 2024. Incident HD patients were defined as patients initiating HD within 60 days of their first-ever maintenance HD for ESKD therapy [9]. The index date was defined as the date of the first maintenance HD session. The eligibility window and exposure‑assessment window were restricted to a 30‑day period surrounding the index date (30 days before and 30 days after the first HD session). Demographic characteristics, laboratory parameters, and vascular‑access types were extracted from records closest to the index date within this 30‑day window. According to local HD quality‑control requirements in China, routine laboratory and screening examinations are generally completed before the first HD initiation. Baseline patient characteristics included age, sex, body mass index, medical history, Charlson Comorbidity Index score, and laboratory parameters. Documentation of any past or present medical condition in the medical record was deemed sufficient for diagnosis. Inclusion criteria were incident HD patients who were ≥18 years, received regular HD with 2 to 3 sessions a week and 4 h per session. Patients with a history of peritoneal dialysis or kidney transplantation, those who died within 60 days after the first HD (per our incident‑HD definition), and those with incomplete follow‑up data, were excluded.

### Exposure definition and outcome assessment

In China, few patients initiate HD with an arteriovenous graft (AVG) compared with those using an AVF or CVC; therefore, AVG and AVF were combined as permanent vascular access for analysis in this study [1]. The primary exposure of interest was the vascular access type at HD initiation, with patients categorized into three groups: AVF/AVG, CVC with maturing AVF/AVG (patients who initiated HD *via* a CVC with a maturing AVF/AVG), and CVC-only. The primary outcome was all-cause mortality. The mortality date was extracted from the Chinese Center for Disease Control and Prevention. Survival time was calculated from the first HD session date until death, transplantation, transfer to peritoneal dialysis, the last visit, or April 2024, whichever occurred first.

### Prediction model, propensity score matching, and subgroup analyses

The Cox proportional hazards regression was performed to investigate the associations between vascular access used at HD initiation and patient survival, with AVF/AVG as the reference group. Variables with *p* < 0.1 in univariate analysis and those with clinical‑biological relevance were entered into the multivariable Cox regression model. The proportional hazards assumption was verified *via* Schoenfeld partial residuals. All Continuous covariates were entered in raw continuous form; Martingale‑residual diagnostics were performed and did not indicate prominent non‑linearity. Variance inflation factors (VIF) were calculated to detect multicollinearity; all VIF values were less than 5, suggesting no severe collinearity. To further balance patient characteristics across vascular access groups, propensity score matching (PSM) was additionally performed. Propensity scores were estimated using the same set of baseline covariates included in the multivariable Cox regression model. We implemented 1:1, 1:2, and 1:4 caliper‑based matching, with the caliper width set at 0.2 times the standard deviation of the propensity score. Standardized mean differences (SMDs) were calculated for each covariate before and after matching to assess between‑group balance; an SMD < 0.1 was defined as acceptable balance. Cox proportional hazards regression was repeated among the successfully matched sample to verify the robustness of the primary findings. Subgroup analyses were added to test effect modification stratified by age and sex, while an interaction term was incorporated to evaluate potential effect modification by these subgroup variables.

### Statistical analyses

The *t*-test or Mann–Whitney *U* test was used to compare normally or non-normally distributed continuous variables. The Chi-square test or Fisher’s exact test was used to compare categorical variables, as appropriate. Kaplan–Meier survival analysis was performed to plot survival curves for different vascular access groups, and the log-rank test was adopted for intergroup survival comparisons. Missing rates for individual baseline variables were less than 5% in the overall cohort. Multiple imputation under the missing‑at‑random assumption was performed to handle missing values for analyses. Notably, PSM cannot be implemented on multiply‑imputed datasets; therefore, PSM analyses were restricted to a complete‑case subset without any missing covariate data. P-values were two-tailed and *p* < 0.05 was considered statistically significant. Statistical analysis was performed using R version 4.1.1 (R Foundation for Statistical Computing, Vienna, Austria).

### Ethics approval

The study protocol was approved by the Medical Ethics Committees of Wuhan Central Hospital (approval number: WHZXKYL2022-083) and Nanfang Hospital, Southern Medical University (approval number: NFEC-2019-213). The Medical Ethics Committees waived the requirement for informed consent because this was a retrospective study using de-identified historical clinical data. All research procedures involving human participants were performed in accordance with the Declaration of Helsinki.

## Results

### Baseline characteristics by vascular access type at HD initiation

This study included 16,717 incident HD patients enrolled in the CRDS database from January 2000 to April 2024, with 1,953 from North China, 4,595 from East China, 8,860 from South China, and 1,309 from West China (Supplementary Table S1). Table 1 shows baseline characteristics by vascular access type at HD initiation. The mean age of the study population was 55.62 ± 10.93 years, and 61.54% were male. At HD initiation, 7,865 patients (47.05%) had diabetes mellitus, 2,760 (16.51%) had coronary heart disease, 4,116 (24.62%) had congestive heart failure, and 14,709 (87.99%) had hypertension. Patients in CVC-only group were older, more likely to be female, had more severe comorbidities, and had a higher prevalence of diabetes mellitus, cardiovascular disease, and congestive heart failure, as compared with the AVF/AVG group and the CVC with maturing AVF/AVG group (all *p* < 0.05).

**Table 1.** Baseline characteristics by vascular access type at HD initiation.

Characteristic	Vascular access type			
	AVF/AVG (*n* = 4,876)	CVC with maturing AVF/AVG (*n* = 10,160)	CVC-only (*n* = 1,681)	*p* value
Age, y, mean (SD)	54.26 ± 10.19	55.42 ± 10.95	62.79 ± 11.81	<0.01
Sex, male, n (%)	3,025 (62.04%)	6,421 (63.20%)	842 (50.09%)	<0.01
BMI, kg/m^2^, mean (SD)	24.97 ± 4.77	24.22 ± 4.92	22.64 ± 5.94	0.08
Congestive cardiac failure, n (%)	644 (13.21%)	2,764 (27.20%)	708 (42.12%)	<0.01
Cardiovascular disease, n (%)	488 (10.01%)	1,788 (17.60%)	484 (28.79%)	<0.01
Hypertension, n (%)	4,218 (86.51%)	8,981 (88.40%)	1,510 (89.83%)	0.09
Diabetes mellitus, n (%)	2,136 (43.81%)	4,806 (47.30%)	923 (54.91%)	<0.01
Charlson score, mean (SD)	4.03 ± 2.16	4.56 ± 2.32	5.55 ± 2.58	<0.01
Hemoglobin, g/dL, mean (SD)	8.54 ± 1.41	8.23 ± 1.54	8.20 ± 1.75	<0.01
Albumin, g/dL, mean (SD)	3.54 ± 0.58	3.45 ± 0.58	3.36 ± 0.61	<0.01
Calcium, mmol/l, mean (SD)	2.09 ± 0.24	2.08 ± 0.25	2.09 ± 0.27	0.05
Phosphate, mmol/l, mean (SD)	1.81 ± 0.62	1.79 ± 0.71	1.81 ± 0.74	0.46
Potassium, mmol/l, mean (SD)	4.45 ± 0.98	4.35 ± 0.92	4.48 ± 1.27	<0.01
Intact parathyroid hormone, pg/mL, mean (SD)	346.48 ± 330.67	360.05 ± 358.34	342.48 ± 347.56	0.04
eGFR, mL/min/1.73 m^2^, mean (SD)	6.38 ± 2.13	6.40 ± 2.59	6.04 ± 2.98	0.01

HD, hemodialysis; AVF/AVG, hemodialysis initiation *via* arteriovenous fistula or graft; CVC with maturing AVF/AVG, hemodialysis initiation *via* central venous catheter with maturing arteriovenous fistula or graft; CVC-only, hemodialysis initiation *via* central venous catheter alone; BMI, body mass index (calculated as weight in kilograms divided by height in meters squared); eGFR, estimated glomerular filtration rate; SD, standard deviation.

### Temporal trends and distribution patterns of vascular access among incident HD patients

In this study, 29.17% of patients (4,876 cases) initiated HD with AVF/AVG, whereas CVC remained the most common vascular access in Chinese incident HD patients, accounting for 70.83% (11,841 cases), of which 60.78% (10,160 cases) using CVC with maturing AVF/AVG and 10.06% (1,681 cases) using CVC-only (Supplementary Figure). Figure 1A shows 10-year trends of vascular access use among incident HD patients in China. The proportion of patients initiating HD with AVF/AVG increased markedly from 26.24% in 2015 to 35.77% in 2024. Meanwhile, the proportion of CVC with maturing AVF/AVG declined slightly from 57.60% to 55.96%, while that of CVC-only decreased substantially from 16.16% to 8.27%. Figure 1B shows regional differences in vascular access use. Vascular access use varied substantially across regions. The proportion of patients initiating HD with AVF/AVG was highest in West China (34.37%) and lowest in South China (20.18%). Meanwhile, the proportion of patients using CVC-only was highest in North China (13.52%) and lowest in West China (8.86%). Figure 1C shows age-related differences in vascular access use. The proportion of patients initiating HD with AVF/AVG declined with advancing age, while the proportion of patients using CVC-only increased. Among patients aged <60, 60–80, and >80 years, the proportion initiating HD with AVF/AVG was 30.64%, 27.17%, and 16.32%, respectively, whereas that for CVC-only were 8.54%, 11.74%, and 32.06%, respectively. Figure 1D shows sex differences in vascular access use. The proportion of patients initiating HD with AVF/AVG was comparable between men and women (29.40% vs. 28.79%), whereas CVC-only use at HD initiation was more prevalent in women than in men (13.05% vs. 8.18%).

**Figure 1.**
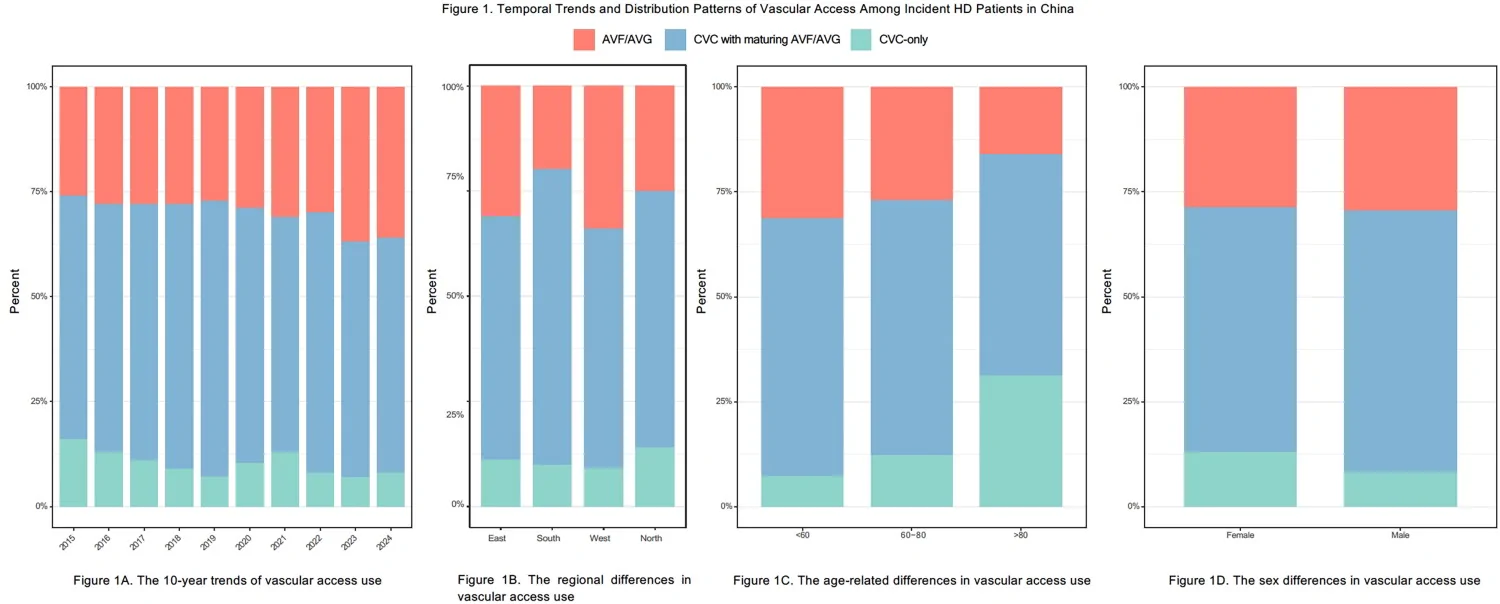
Temporal trends and distribution patterns of vascular access among incident HD patients in China. (A) The 10-year trends of vascular access use. The proportion of patients initiating HD with AVF/AVG increased markedly from 26.24% in 2015 to 35.77% in 2024. Meanwhile, the proportion of CVC with maturing AVF/AVG declined slightly from 57.60% to 55.96%, while that of CVC-only decreased substantially from 16.16% to 8.27%. (B) The regional differences in vascular access use. The proportion of patients initiating HD with AVF/AVG was highest in West China (34.37%) and lowest in South China (20.18%). Conversely, the proportion of patients using CVC-only was lowest in West China (8.86%) and highest in North China (13.52%). (C) The age-related differences in vascular access use. Among patients aged <60, 60–80, and >80 years, the proportion initiating HD with AVF/AVG were 30.64%, 27.17%, and 16.32%, respectively, whereas those for CVC-only were 8.54%, 11.74%, and 32.06%, respectively. (D) The sex differences in vascular access use. The proportion of patients initiating HD with AVF/AVG was comparable between men and women (29.40% vs. 28.79%), whereas CVC-only use at HD initiation was more prevalent in women than in men (13.05% vs. 8.18%). Abbreviations: HD, hemodialysis; AVF/AVG, hemodialysis initiation via arteriovenous fistula or graft; CVC with maturing AVF/AVG, hemodialysis initiation via central venous catheter with maturing arteriovenous fistula or graft; CVC-only, hemodialysis initiation via central venous catheter alone.

### Vascular access at HD initiation and patient survival

Among 16,717 patients during a mean follow-up of 64.41 months, a total of 5,813 patients died, including 1,406 in the AVF/AVG group, 3,532 in the CVC with maturing AVF/AVG group, and 875 in the CVC-only group. As shown in Figure 2, the overall mortality rate was 6.57 (95% CI 6.40–6.75) per 100 person-years, and the rates for the AVF/AVG, CVC with maturing AVF/AVG, and CVC-only groups were 4.15 (95% CI 3.93–4.38), 7.24 (95% CI 7.00–7.49), and 15.08 (95% CI 14.07–16.15) per 100 person-years, respectively. Kaplan–Meier survival curves for all-cause mortality were plotted according to vascular access type at HD initiation, as shown in Figure 3. In both the overall population and after stratification by age and sex, all-cause mortality was higher in the CVC-only and CVC with maturing AVF/AVG groups than in the AVF/AVG group by log-rank test (all *p* < 0.05).

**Figure 2.**
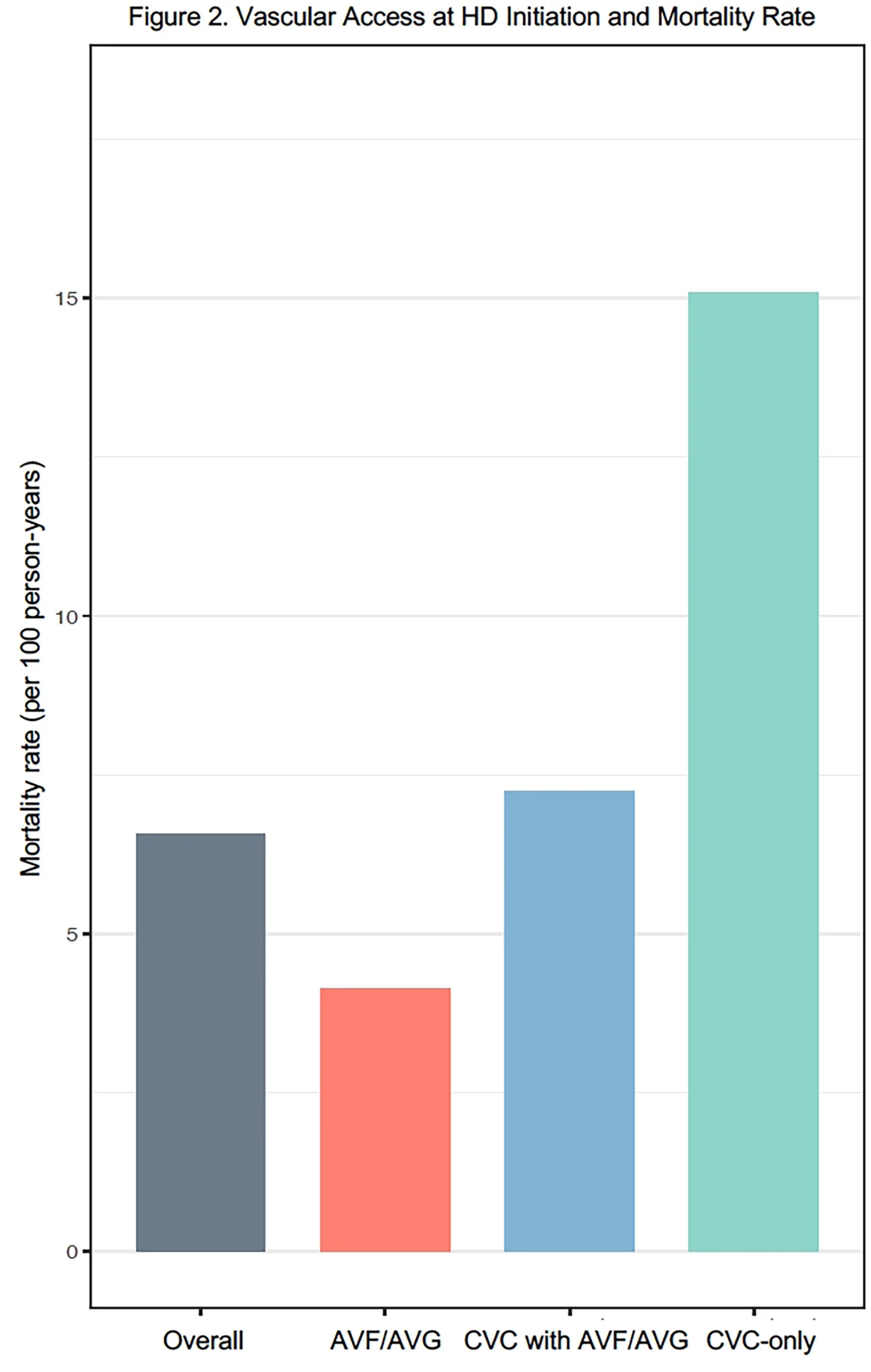
Vascular access at HD initiation and mortality rate. The overall mortality rate was 6.57 (95% CI 6.40–6.75) per 100 person-years, and the rates for the AVF/AVG, CVC with maturing AVF/AVG, and CVC-only groups were 4.15 (95% CI 3.93–4.38), 7.24 (95% CI 7.00–7.49), and 15.08 (95% CI 14.07–16.15) per 100 person-years, respectively. Abbreviations: HD, hemodialysis; AVF/AVG, hemodialysis initiation via arteriovenous fistula or graft; CVC with AVF/AVG, hemodialysis initiation via central venous catheter with maturing arteriovenous fistula or graft; CVC-only, hemodialysis initiation via central venous catheter alone.

**Figure 3.**
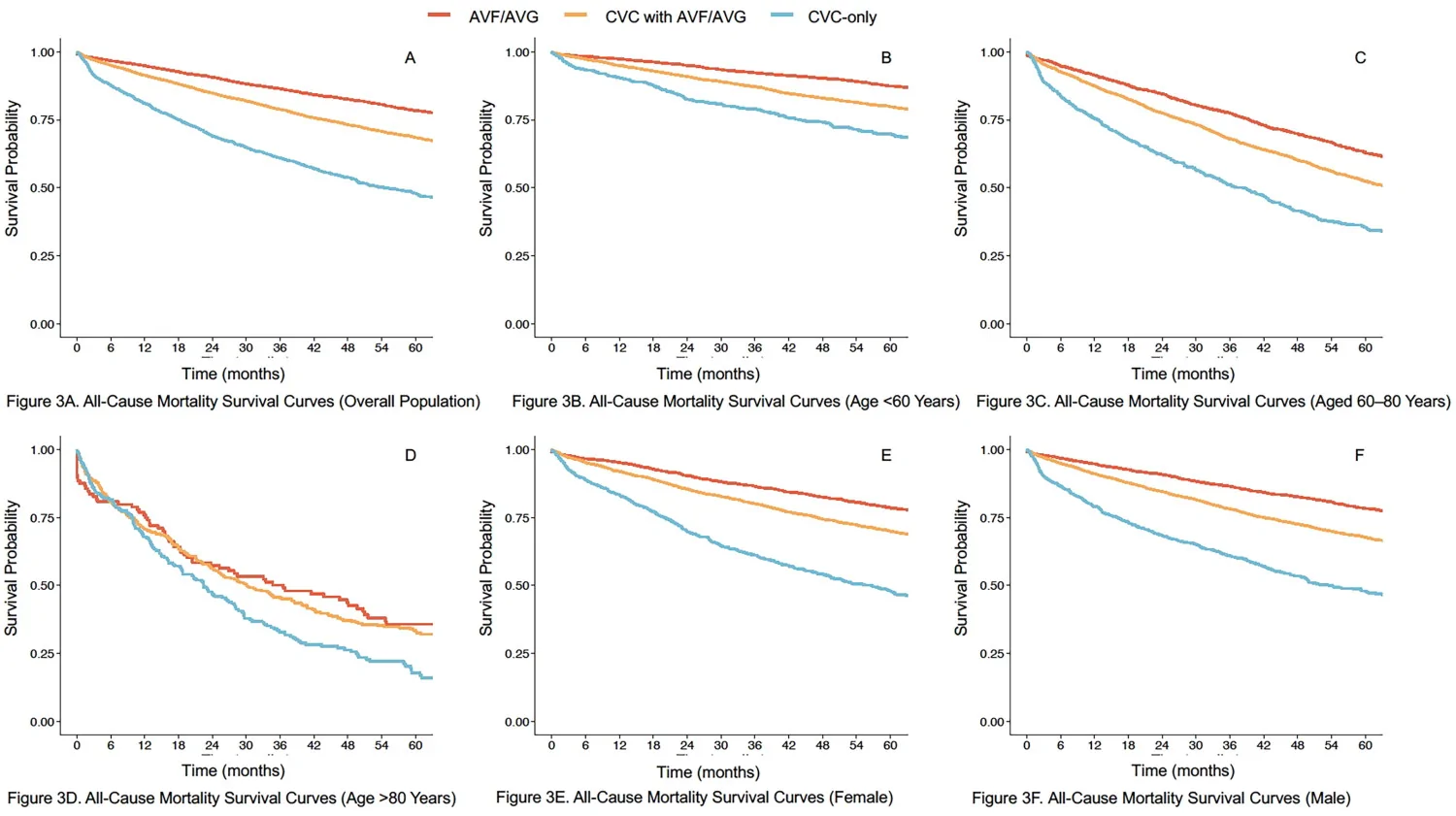
Survival curves for all-cause mortality according to vascular access at HD initiation. (A) All-cause mortality survival curves (overall population). (B) All-cause mortality survival curves (age <60 years). (C) All-cause mortality survival curves (aged 60–80 years). (D) All-cause mortality survival curves (age >80 years). (E) All-cause mortality survival curves (female). (F) All-cause mortality survival curves (male). In both the overall population and after stratification by age and sex, all-cause mortality was higher in the CVC-only group than in the AVF/AVG and CVC with maturing AVF/AVG groups by log-rank test (all *p* < 0.05). Abbreviations: HD, hemodialysis; AVF/AVG, hemodialysis initiation via arteriovenous fistula or graft; CVC with maturing AVF/AVG, hemodialysis initiation via central venous catheter with maturing arteriovenous fistula or graft; CVC-only, hemodialysis initiation via central venous catheter alone.

In the unadjusted Cox proportional hazards regression model, patients in both the CVC with maturing AVF/AVG group (HR = 1.52, 95% CI 1.43–1.63) and the CVC-only group (HR = 3.03, 95% CI 2.77–3.31) had an increased all-cause mortality risk compared with those in the AVF/AVG group (Table 2). After adjusting for confounding variables including age, sex, Charlson comorbidity score and laboratory parameters, multivariable Cox proportional hazards regression analysis revealed that although the association was slightly attenuated, patients in the CVC with maturing AVF/AVG group (HR = 1.36, 95% CI 1.27–1.45) and the CVC-only group (HR = 1.91, 95% CI 1.74–2.01) still had a higher risk of all-cause mortality compared with those in the AVF/AVG group (Table 2). Propensity scores were calculated within the complete‑case subset to match AVF/AVG patients with CVC patients (including CVC with maturing AVF/AVG and CVC-only patients) at 1:1, 1:2, and 1:4 ratios, respectively. A subset of 3,258 patients with complete data was included, comprising 362 patients in the AVF/AVG group, 1,448 patients in the CVC with maturing AVF/AVG group, and 1,448 patients in the CVC‑only group (Supplementary Figure). To evaluate covariate balance after PSM, SMD values for all included baseline variables were compared before and after matching. All covariates achieved SMD values below 0.1 after 1:1, 1:2 and 1:4 matching, suggesting satisfactory balance of patient characteristics across groups (Supplementary Table S2). The HRs derived from Cox models based on each matched dataset were consistent with the results from the primary unmatched analysis (Figure 4).

**Figure 4.**
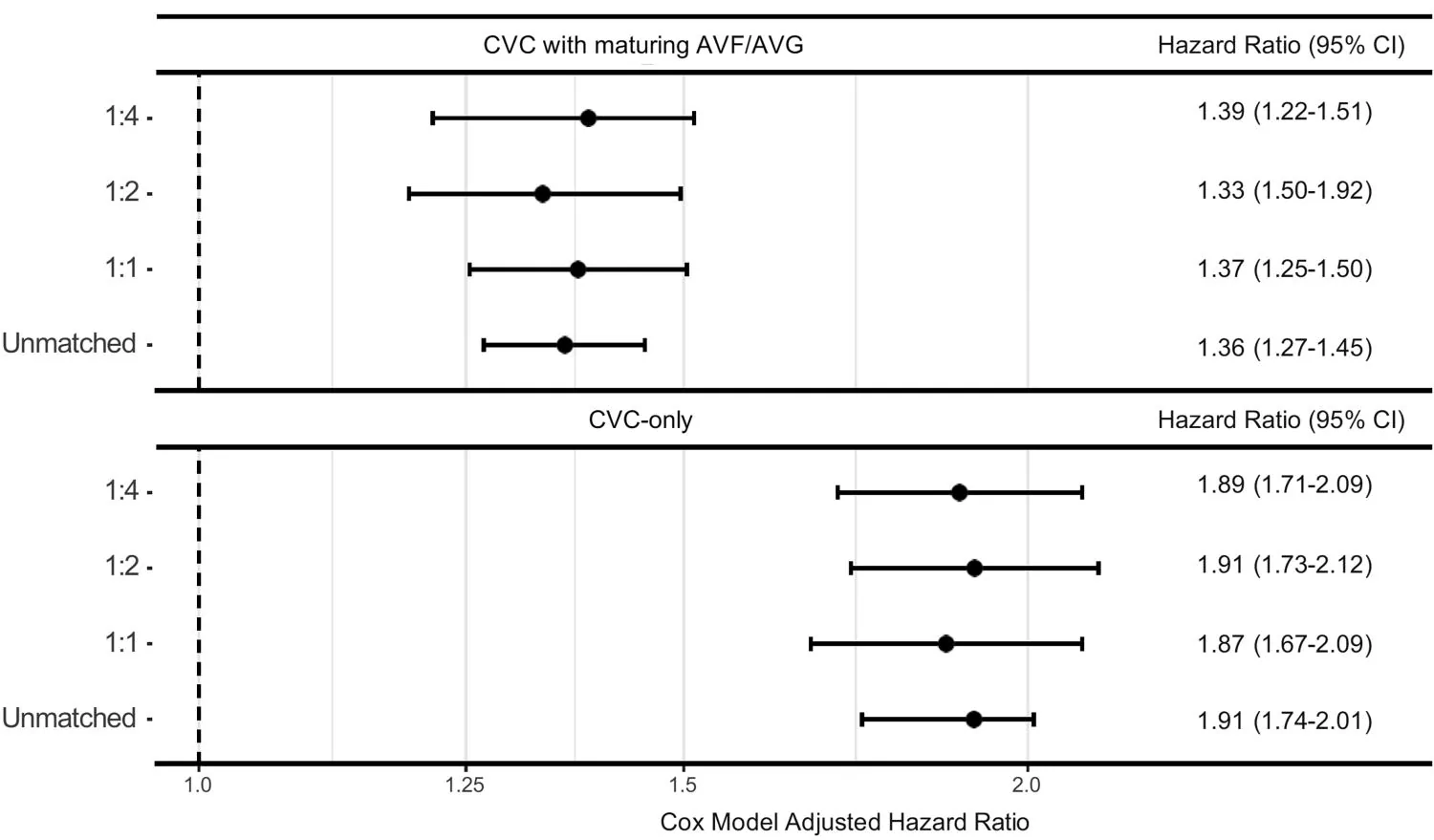
Effect of matching methods on hazard ratios. Propensity scores were generated using all available patient characteristics to match AVF/AVG patients with CVC patients (including CVC with maturing AVF/AVG and CVC-only) at 1:1, 1:2, and 1:4 ratios, respectively. A subset of 3,258 patients with complete data were included, comprising 362 AVF/AVG patients and 2,896 CVC patients (1,448 patients in the CVC with maturing AVF/AVG group and 1,448 patients in the CVC‑only group). The Cox proportional hazards regression model performed previously was repeated, yielding HRs and 95% CIs consistent with the main results. Abbreviations: AVF/AVG, hemodialysis initiation via arteriovenous fistula or graft; CVC with maturing AVF/AVG, hemodialysis initiation via central venous catheter with maturing arteriovenous fistula or graft; CVC-only, hemodialysis initiation via central venous catheter alone; HR, hazard ratio; CI, confidence interval.

**Table 2.** Mortality risk based on cox proportional hazards model.

Characteristic	Univariable		Multivariable	
Vascular access type				
CVC with maturing AVF/AVG	1.52, 1.43–1.63,	<0.01	1.36, 1.27–1.45	<0.01
CVC-only	3.03, 2.77–3.31	<0.01	1.91, 1.74–2.01	<0.01
Age, y, mean (SD)	1.05, 1.04–1.05	<0.01	1.04, 1.01–1.06	<0.01
Sex, male, n (%)	1.08, 1.02–1.14	0.01	1.01, 0.96–1.07	0.63
BMI, kg/m^2^, mean (SD)	0.96, 0.92–0.99	0.01	0.97, 0.95–0.98	0.03
Congestive cardiac failure, n (%)	1.93, 1.82–2.04	<0.01	1.26, 1.18–1.34	<0.01
Cardiovascular disease, n (%)	2.30, 2.17–2.45	<0.01	1.25, 1.17–1.33	<0.01
Hypertension, n (%)	1.38, 1.26–1.52	<0.01	1.11, 1.02–1.21	0.04
Diabetes mellitus, n (%)	1.78, 1.69–1.88	<0.01	1.30, 1.22–1.38	<0.01
Charlson score, mean (SD)	1.29, 1.28–1.30	<0.01	1.03, 1.01–1.04	<0.01
Hemoglobin, g/dL, mean (SD)	0.89, 0.82–0.96	<0.01	0.90, 0.84–0.97	<0.01
Albumin, g/dL, mean (SD)	0.96, 0.93–0.99	<0.01	0.97, 0.96–0.98	<0.01
Calcium, mmol/l, mean (SD)	1.26, 1.06–1.48	<0.01	1.18, 1.05–1.33	<0.01
Phosphate, mmol/l, mean (SD)	1.30, 1.17–1.43	<0.01	1.19, 1.06–1.34	<0.01
Potassium, mean (SD), mmol/l	1.14, 1.01–1.17	<0.01	1.10, 1.03–1.15	<0.01
Intact parathyroid hormone, pg/mL, mean (SD)	1.08, 1.02–1.13	<0.01	1.02, 1.01–1.04	<0.01
eGFR, mL/min/1.73 m^2^, mean (SD)	0.81, 0.72–0.90	<0.01	0.87, 0.80–0.95	<0.01

HD, hemodialysis; AVF/AVG, hemodialysis initiation *via* arteriovenous fistula or graft; CVC with maturing AVF/AVG, hemodialysis initiation *via* central venous catheter with maturing arteriovenous fistula or graft; CVC-only, hemodialysis initiation *via* central venous catheter alone; BMI, body mass index (calculated as weight in kilograms divided by height in meters squared); eGFR, estimated glomerular filtration rate; SD, standard deviation.

### Subgroup analyses

All-cause mortality risk according to vascular access type at HD initiation in the overall population and subgroups is presented in Table 3. In both the CVC with maturing AVF/AVG group and CVC-only group, no significant sex-related differences were observed in all-cause mortality risk (HRs for men and women: 1.38 vs. 1.23; 2.05 vs. 1.81, respectively), with all P-values for interaction > 0.05. In the CVC with maturing AVF/AVG group, patients aged >80 years demonstrated no increased mortality risk (HR 1.13, 95% CI 0.86–1.47, *p* = 0.38) compared with those aged <60 years (HR 1.62, 95% CI 1.47–1.79, *p* < 0.01) and 60–80 years (HR 1.37, 95% CI 1.25–1.50, *p* < 0.01). In the CVC-only group, patients aged > 80 years exhibited a reduced risk of mortality (HR 1.42, 95% CI 1.04–1.92, *p* < 0.01) compared with those aged < 60 years (HR 2.66, 95% CI 2.27–3.13, *p* < 0.01) and 60–80 years (HR 1.98, 95% CI 1.72–2.27, *p* < 0.01).

**Table 3.** All-cause mortality risk in the overall population and subgroups.

Characteristic	AVF/AVG as reference	
	CVC with maturing AVF/AVG	CVC-only
Overall	1.36, 1.27–1.45	1.91, 1.74–2.01
Sex		
Male	1.38, 1.25–1.51, <0.01	2.05, 1.70–2.46, <0.01
Female	1.23, 1.08–1.39, <0.01	1.81, 1.43–2.24, <0.01
Age		
<60	1.62, 1.47–1.79, <0.01	2.66, 2.27–3.13, <0.01
60–80	1.37, 1.25–1.50, <0.01	1.98, 1.72–2.27, <0.01
>80	1.13, 0.86–1.47, 0.38	1.42, 1.04–1.92, <0.01

AVF/AVG, hemodialysis initiation *via* arteriovenous fistula or graft; CVC with maturing AVF/AVG, hemodialysis initiation *via* central venous catheter with maturing arteriovenous fistula or graft; CVC-only, hemodialysis initiation *via* central venous catheter alone.

## Discussion

While vascular access use has improved substantially among prevalent HD patients, such progress has been much slower among incident HD patients [9]. Although numerous studies have reported higher mortality and hospitalization rates associated with CVC use in incident HD patients, and importantly, lower first-year mortality among patients who converted from CVC to AVF/AVG [20,21], a US Renal Data System-based study including more than 500,000 patients revealed that 14.0% initiated HD with AVF, 3.4% with AVG, and 82.5% with CVC [22]. According to our data among Chinese incident HD patients, during the period from 2000 to 2024, 29.17% initiated HD *via* AVF/AVG, whereas CVC remained the predominant vascular access type at 70.83%, including 60.78% *via* CVC with maturing AVF/AVG and 10.06% *via* CVC-only. Over the past decade, the proportion of patients initiating HD with AVF/AVG rose markedly from 26.24% in 2015 to 35.77% in 2024. Meanwhile, the proportion of CVC with maturing AVF/AVG declined slightly from 57.60% to 55.96%, whereas CVC-only decreased substantially from 16.16% to 8.27%, reflecting the ongoing efforts of Chinese dialysis service providers, the National Hemodialysis Quality Control Center, and the China Clinical Practice Guidelines for Vascular Access to optimize vascular access use among incident HD patients. Allon et al. proposed that the current US reimbursement system is a major factor underlying the continued high level of CVC use for US incident HD patients [23]. Antiquated healthcare policies prevent patients and healthcare providers from receiving adequate reimbursement for the timely creation of optimal HD access, thereby resulting in an unnecessary economic burden on the US healthcare system and unwarranted risks to patients’ lives. In contrast, China’s reimbursement system enables patients to receive insurance coverage for AVF/AVG surgery prior to initiating HD [1]. Previous studies have shown that CVC-based HD initiation is the result of patients not being referred in time to the nephrologist [24]. With the gradual improvement of chronic kidney disease (CKD) clinics, coupled with early detection for CKD and timely referral to nephrologists, more patients are able to undergo AVF/AVG creation in advance, thereby avoiding the use of CVC at HD initiation [12]. Furthermore, in China, AVF/AVG creation is performed by nephrologists, which eliminates delays associated with referral to surgeons, thereby contributing to a high prevalence of AVF/AVG use among incident HD patients.

In this study, the vascular access distribution among Chinese incident HD patients was similar to that among prevalent HD patients in many aspects. In the Chinese Dialysis Outcomes and Practice Patterns Study (DOPPS), AVF was the predominant vascular access type among prevalent HD patients, accounting for 88.2% [1]. In our study, 89.94% of incident HD patients had established AVF/AVG. Of these patients, 29.17% used AVF/AVG as their initial access and 60.78% initiated HD with CVC but had a maturing AVF/AVG. Among Chinese prevalent HD patients, AVF use was higher in younger patients, males, and those with fewer comorbidities [1], consistent with our findings in Chinese incident HD patients: AVF/AVG was more common in younger patients (<60, 60–80, >80: 30.64%, 27.17%, 16.32%), while CVC-only was more prevalent in elderly patients (<60, 60–80, >80: 8.52%, 11.74%, 32.06%); the proportion of AVF/AVG as initial vascular access was similar between males and females (29.40% vs. 28.79%), whereas CVC-only was more prevalent in females (8.18% vs. 13.05%); patients with CVC-only as their initial vascular access had a higher Charlson Comorbidity Index score than those with CVC with maturing AVF/AVG and those with AVF/AVG (5.55 ± 2.58, 4.56 ± 2.32, and 4.03 ± 2.16, respectively). Prior exposure to CVC may influence patients’ vascular access preference [25]. Therefore, avoiding initial CVC use at HD initiation in incident HD patients may help reduce CVC utilization among prevalent HD patients. This may explain the higher AVF use in the Chinese DOPPS cohort compared with many other DOPPS-participating countries [1,9].

As pointed out by the recent KDOQI Vascular Access Guideline, the current quality of evidence linking initial vascular access type and patient outcomes is suboptimal and not sufficient to make recommendations [4]. Reports from DOPPS and large‑scale national registries across North America, Europe and Asia have illustrated that regional differences may shape vascular‑access utilization and subsequent patient prognosis [2,7,9]. Cumulative evidence from international observational cohorts highlights consistent excess risks of infection, thrombosis, and access failure for patients initiating HD with CVC relative to AVF/AVG, even after adjustment for baseline comorbidities [3–5]. While many Western cohorts have reported high risks of mortality for CVC‑dependent incident HD patients, real‑world data from Asian populations remain relatively limited, especially nationwide data describing incident‑access patterns and associated long‑term outcomes [1]. Our real‑world findings from the CRDS database add complementary evidence for Asian ESKD populations regarding practice patterns and survival correlates of initial HD access. Our findings indicate that among Chinese incident HD patients, initiating dialysis using CVC with a maturing AVF/AVG or CVC alone exhibited 36% and 91% higher all-cause mortality risks, respectively, compared with those using AVF/AVG, which is consistent with findings from populations in other countries [22,26–28]. Although initiating HD *via* CVC with maturing AVF/AVG attenuated mortality risk, this may be attributable to earlier conversion from CVC to AVF/AVG, thereby avoiding prolonged CVC exposure [29]. Of note, many studies have reported superior dialysis adequacy among patients with AVF/AVG relative to those using CVC, which may serve as one potential mechanism explaining the prognostic differences across vascular‑access groups [30,31]. Nevertheless, Kt/V data are not available in the CRDS database for most participants; hence this hypothesis cannot be directly verified in our present cohort. We also hypothesize that the excess mortality risk among patients initiating HD with CVC may be partially attributable to catheter‑related infections and cardiovascular events. However, cause‑of‑death codes are unavailable in our dataset, so we cannot directly verify which cause of death primarily accounts for the observed risk disparity. In the present study, the large sample size ensured accurate estimation of the benefits associated with AVF/AVG. Meanwhile, we adjusted for numerous demographic and clinical factors that may cause selection bias and confounding *via* multivariate regression models and matching methods, further validating the robustness of our findings. Therefore, consistent with previous studies [32,33], we believe that the survival benefit associated with initiating HD with AVF/AVG may not be attributable to patient selection bias, and the poorer survival in patients initiating HD with CVC may also not be due to severe comorbidities. It is generally considered that patients initiating HD *via* a CVC have significantly more comorbidities and are at prohibitive surgical risk. In this study, we successfully matched each AVF/AVG patient with one or more CVC patients with comparable baseline characteristics, suggesting that many CVC patients could have been candidates for AVF/AVG placement. Furthermore, 66.78% of patients initiated HD with a CVC while having a maturing AVF/AVG, indicating that these patients were suitable surgical candidates; however, delayed creation of AVF/AVG prevented its timely use, resulting in a significantly increased risk of mortality. This finding underscores the need for timely referral for AVF/AVG creation to ensure a mature AVF/AVG at HD initiation.

In contrast to findings from another large cohort study [28] suggesting that the higher mortality risk associated with initiating HD *via* CVC was more pronounced in men than in women, subgroup analyses in our study showed no significant sex difference in all‑cause mortality among Chinese incident HD patients who used CVC as their initial vascular access. Age-stratified subgroup analyses showed that the magnitude of increased mortality risk in the CVC-only group gradually decreased with advancing age (<60, 60–80, and >80 years; HRs: 2.66, 1.98, and 1.42, respectively), whereas no increased mortality risk was observed in patients aged > 80 years in the CVC with maturing AVF/AVG group (*p* > 0.05). This may be due to the relatively short life expectancy of elderly patients, which reduces or prevents the survival benefit conferred by AVF/AVG from being observed [34,35]. These findings suggest that, in clinical practice, super‑elderly patients may initiate dialysis with CVC and subsequently convert to AVF/AVG, without apparent elevation in mortality risk in this observational cohort. These results complement existing evidence supporting an individualized, patient‑centered approach to determining optimal vascular access across different patient populations.

Compared with other studies, our study has the advantage of a large patient population that is representative of the incident HD patients in China with reliable and detailed data, and we employed PSM and multivariate regression models to minimize selection bias and confounding factors. To the best of our knowledge, this is the first study to comprehensively elucidate the temporal trends and distribution patterns of initial vascular access, as well as its association with mortality, among incident HD patients in China. Several limitations should be acknowledged. First, this was a retrospective observational study. Owing to potential residual confounding, only statistical associations, rather than causal relationships, can be inferred from our analyses, even after multivariate adjustment and PSM. Residual confounding may include granularity of comorbidity severity, nephrology referral timing, and insurance coverage, all of which may be associated with initial vascular access selection and subsequent mortality. Dialysis centers with a higher proportion of AVF placement may also adopt other clinical practices that improve patient survival, which could introduce confounding bias. Second, owing to market, economic and insurance coverage factors [1], the number of patients with AVG as the initial HD access was relatively small compared with AVF or CVC. We therefore combined AVF and AVG as permanent vascular access for analysis, making it difficult to conduct an accurate comparison of AVG alone. Third, information regarding vascular‑access conversion during follow‑up was unavailable in the present study. And the database records CVC as a single category and cannot further distinguish non‑tunneled temporary CVC from tunneled long‑term CVC. These two CVC subtypes were combined in our analysis, despite well‑documented differences in infection and complication risks. Multiple vascular‑access transitions may occur during follow‑up, such as switching from non‑tunneled CVC to tunneled long‑term CVC or functional AVF/AVG. Under our initial‑exposure analytical framework, patients starting HD with CVC but later receiving permanent access remained categorized in the CVC group. Such misclassification may introduce bias toward the null and dilute the observed mortality hazard related to initial CVC use, which suggests that our reported hazard ratios may underestimate the genuine mortality risk of initiating HD with CVC. Fourth, we could not perform stratified analyses by participating center or calendar time. Data from individual centers were uploaded sequentially into the CRDS database, with substantial heterogeneity in patient volume across sites; many centers contained only a small number of patients and were unsuitable for separate stratified modeling. Furthermore, limited electronic‑record infrastructure in many centers in earlier years resulted in sparse historical data for the pre‑2014 period. Therefore, our primary Cox model did not explicitly adjust for inter‑center heterogeneity or within‑center clustering effects. Fifth, data quantifying dialysis adequacy (e.g. Kt/V) were largely unavailable in the CRDS database. We could not evaluate whether differences in dialysis adequacy mediated the survival disparities observed across different vascular‑access groups. Finally, only all‑cause mortality and corresponding date of death were available from CRDS linked CDC records. Detailed coded cause‑of‑death information was not captured in this database. As a result, we were unable to conduct cause‑specific mortality analyses to distinguish cardiovascular death, infection‑related death, or other causes of death across vascular access groups.

## Conclusions

This nationwide study demonstrates ongoing and considerable improvements in vascular access practice in China. Over the 25-year period from 2000 to 2024, the initial vascular access among incident HD patients in China gradually shifted from CVC to AVF/AVG. This trend was most noticeable in East China and West China, while elderly patients and females were more likely to initiate HD *via* CVC. Among incident HD patients, CVC use at HD initiation showed a statistical association with elevated all‑cause mortality risk relative to AVF/AVG, although the presence of a maturing AVF/AVG appeared to attenuate this observed risk in our cohort. These results support the idea that AVF/AVG should be created earlier in patients with ESKD to avoid CVC use at HD initiation and thereby improve long-term survival. This study helps to better understand the current status of HD management in China, identifies areas for improvement, and explores more appropriate dialysis practice patterns for Chinese HD patients. We also anticipate that with the advancement of ongoing and future clinical studies, more high-quality data will be generated to further improve HD clinical practice patterns in China.

## Supplementary Material

Supplementary Figure.jpg

Supplementary Table S2.docx

Supplementary Table S1.docx

## Data Availability

Currently, CRDS data is only free to share with participating collaborators who have signed the cooperation agreement. If you are willing to cooperate, please contact the CRDS database administrator *via* the official email address: ncrckd@163.com (http://www.crds-network.org.cn/#/joinUs/joinus). The statistical analysis code for this study is available from the corresponding author upon reasonable request.
