# Supplementary material for: Trends in incident hemodialysis access and mortality in China: results from China Renal Data System 2000–2024
Source: Ren Fail. 2026 Oct 25;48(1):2747546. doi: 10.1080/0886022X.2026.2747546 (PMC13700462; doi:10.1080/0886022X.2026.2747546)
Supplement: Supplementary file 1 [file IRNF_A_2747546_SM6241.docx]

| **Supplementary Table S1.** Sex‑ and age‑stratified distribution of initial vascular access in the primary cohort | | | | |
| --- | --- | --- | --- | --- |
|  | AVF/AVG  (numerators [proportions]) | CVC with maturing AVF/AVG  (numerators [proportions]) | CVC-only  (numerators [proportions]) | Total  (denominators) |
| Overall | 4,876 (29.17%) | 1,0160 (60.78%) | 1,681 (10.06%) | 16,717 (100%) |
| Male | 3,025 (29.40%) | 6,421 (62.41%) | 842 (8.18%) | 10,288 (100%) |
| Female | 1,851 (28.79%) | 3,739 (58.16%) | 839 (13.05%) | 6,429 (100%) |
| Age < 60 | 2,977 (30.64%) | 5,909 (60.82%) | 830 (8.54%) | 9,716 (100%) |
| Age 60 - 80 | 1,624 (27.17%) | 3,652 (61.09%) | 702 (11.74%) | 5,978 (100%) |
| Age >80 | 167 (16.32%) | 528 (51.61%) | 328 (32.06%) | 1,023 (100%) |
| AVF/AVG, arteriovenous fistula or graft; CVC, central venous catheter.  Values are presented as numerators (proportions, %) within each stratum. Denominators represent the total number of patients in each corresponding subgroup from the primary analytical dataset. | | | | |
