# Supplementary material for: Trends in incident hemodialysis access and mortality in China: results from China Renal Data System 2000–2024
Source: Ren Fail. 2026 Oct 25;48(1):2747546. doi: 10.1080/0886022X.2026.2747546 (PMC13700462; doi:10.1080/0886022X.2026.2747546)
Supplement: Supplementary file 2 [file IRNF_A_2747546_SM6242.docx]

**Table legend.**

Propensity‑score matching (PSM) was performed within a complete‑case subset without missing covariate data. Baseline covariates included age, sex, body mass index, comorbid conditions, Charlson comorbidity index score, hemoglobin, albumin, serum calcium, serum phosphate, serum potassium, intact parathyroid hormone, and estimated glomerular filtration rate (eGFR). PSM was performed with 1:1, 1:2, and 1:4 caliper-based matching schemes; caliper width was set to 0.2 times the standard deviation of the propensity score. Standardized mean difference (SMD) < 0.1 indicates acceptable between-group covariate balance. Table S2a shows the pairwise comparison between AVF/AVG and CVC with maturing AVF/AVG; Table S2b shows the pairwise comparison between AVF/AVG and CVC-only.

| **Supplementary Table S2a.** SMDs of baseline covariates before and after PSM (AVF/AVG vs. CVC with maturing AVF/AVG) | | | | |
| --- | --- | --- | --- | --- |
| Covariate | Before matching | After 1:1 matching | After 1:2 matching | After 1:4 matching |
| Age | 0.11 | 0.04 | 0.05 | 0.06 |
| Gender (male) | 0.02 | 0.01 | 0.02 | 0.02 |
| BMI | 0.15 | 0.04 | 0.04 | 0.05 |
| Congestive cardiac failure | 0.35 | 0.03 | 0.04 | 0.05 |
| Cardiovascular disease | 0.22 | 0.03 | 0.04 | 0.04 |
| Hypertension | 0.05 | 0.02 | 0.02 | 0.03 |
| Diabetes mellitus | 0.07 | 0.02 | 0.03 | 0.03 |
| Charlson Comorbidity Index | 0.23 | 0.04 | 0.04 | 0.05 |
| Hemoglobin | 0.21 | 0.03 | 0.04 | 0.05 |
| Albumin | 0.15 | 0.03 | 0.04 | 0.04 |
| Calcium | 0.04 | 0.02 | 0.02 | 0.02 |
| Phosphate | 0.03 | 0.01 | 0.02 | 0.02 |
| Potassium | 0.10 | 0.03 | 0.03 | 0.04 |
| Intact parathyroid hormone | 0.03 | 0.02 | 0.02 | 0.02 |
| eGFR | 0.08 | 0.01 | 0.01 | 0.01 |

| **Supplementary Table S2b.** SMDs of baseline covariates before and after PSM (AVF/AVG vs. CVC-only) | | | | |
| --- | --- | --- | --- | --- |
| Covariate | Before matching | After 1:1 matching | After 1:2 matching | After 1:4 matching |
| Age | 0.77 | 0.04 | 0.05 | 0.06 |
| Gender (male) | 0.24 | 0.03 | 0.04 | 0.04 |
| BMI | 0.43 | 0.04 | 0.04 | 0.05 |
| Congestive cardiac failure | 0.68 | 0.04 | 0.04 | 0.05 |
| Cardiovascular disease | 0.48 | 0.03 | 0.04 | 0.05 |
| Hypertension | 0.10 | 0.02 | 0.03 | 0.03 |
| Diabetes mellitus | 0.22 | 0.03 | 0.03 | 0.04 |
| Charlson Comorbidity Index | 0.63 | 0.04 | 0.05 | 0.06 |
| Hemoglobin | 0.21 | 0.03 | 0.04 | 0.04 |
| Albumin | 0.30 | 0.03 | 0.04 | 0.05 |
| Calcium | 0.01 | 0.01 | 0.01 | 0.02 |
| Phosphate | 0.01 | 0.01 | 0.01 | 0.01 |
| Potassium | 0.02 | 0.01 | 0.01 | 0.02 |
| Intact parathyroid hormone | 0.01 | 0.01 | 0.01 | 0.01 |
| eGFR | 0.13 | 0.02 | 0.03 | 0.03 |
